# Addressing child health inequity through case management of under-five malaria in Nigeria: an extended cost-effectiveness analysis

**DOI:** 10.1186/s12936-022-04113-w

**Published:** 2022-03-09

**Authors:** Rishav Raj Dasgupta, Wenhui Mao, Osondu Ogbuoji

**Affiliations:** 1 Trinity College of Arts and Sciences, Duke University, Durham, NC USA; 2 Center for Policy Impact in Global Health at Duke Global Health Institute, Durham, NC USA; 3 Duke Margolis Center for Health Policy, Durham, NC USA

**Keywords:** Child health inequality, Decision-tree model, Out-of-pocket expenditure, Catastrophic health expenditure, Financial risk protection

## Abstract

**Background:**

Under-five malaria in Nigeria is a leading cause of global child mortality, accounting for 95,000 annual child deaths. High out-of-pocket medical expenditure contributes to under-five malaria mortality by discouraging care-seeking and use of effective anti-malarials in the poorest households. The significant inequity in child health outcomes in Nigeria stresses the need to evaluate the outcomes of potential interventions across socioeconomic lines.

**Methods:**

Using a decision tree model, an extended cost-effectiveness analysis was done to determine the effects of subsidies covering the direct and indirect costs of case management of under-five malaria in Nigeria. This analysis estimates the number of child deaths averted, out-of-pocket (OOP) expenditure averted, cases of catastrophic health expenditure (CHE) averted, and cost of implementation. An optimization analysis was also done to determine how to optimally allocate money across wealth groups using different combinations of interventions.

**Results:**

Fully subsidizing direct medical, non-medical, and indirect costs could annually avert over 19,000 under-five deaths, 8600 cases of CHE, and US$187 million in OOP spending. Per US$1 million invested, this corresponds to an annual reduction of 76 under-five deaths, 34 cases of CHE, and over US$730,000 in OOP expenditure. Due to low initial treatment coverage in poorer socioeconomic groups, health and financial-risk protection benefits would be pro-poor, with the poorest 40% of Nigerians accounting for 72% of all deaths averted, 55% of all OOP expenditure averted, and 74% of all cases of CHE averted. Subsidies targeted to the poor would see greater benefits per dollar spent than broad, non-targeted subsidies. In an optimization scenario, the strategy of fully subsidizing direct medical costs would be dominated by a partial subsidy of direct medical costs as well as a full subsidy of direct medical, nonmedical, and indirect costs.

**Conclusion:**

Subsidizing case management of under-five malaria for the poorest and most vulnerable would reduce illness-related impoverishment and child mortality in Nigeria while preserving limited financial resources. This study is an example of how focusing a targeted policy-intervention on a single, high-burden disease can yield large health and financial-risk protection benefits in a low and middle-income country context and address equity consideration in evidence-informed policymaking.

**Supplementary Information:**

The online version contains supplementary material available at 10.1186/s12936-022-04113-w.

## Background

Despite significant progress in global malaria control over the last two decades, malaria remains one of the leading causes of morbidity and mortality in children under the age of five, who account for two-thirds of the global malaria burden [1, 2]. Recent trends also indicate that progress in malaria control is slowing in the highest burden countries [3]. In Nigeria, which bears 25% of global morbidity, malaria annually accounts for an estimated 60% of outpatient hospital visits, 50 million cases, and 100,000 deaths [4–6]. The most vulnerable Nigerians are under-five children, who experience an average of 2–4 episodes per year and account for as much as 90% of national malaria mortality [7, 8]. Resultingly, as much as 36% of under-five mortality in Nigeria is attributable to malaria [9, 10].

While prompt and effective treatment of malaria has good clinical outcomes in under-fives, cases where treatment is absent, delayed, or ineffective can become severe and lead to life-threatening complications [7]. Nigeria’s high under-five malaria mortality is largely attributable to a health financing system that leaves many individuals uninsured, resulting in high out-of-pocket (OOP) medical expenditure that discourages care-seeking behaviour, especially among the poor (Box 1) [11]. Nigeria has one of the lowest rates of care-seeking for suspected cases of under-five malaria in the world, with just under 20% of all under-fives with fever being brought to health facilities for clinical consultation and parasitological testing [1].

Even when care is sought, the most effective malaria treatments are prohibitively costly and used by few Nigerians [12, 13]. Artemisinin-based combination therapy (ACT), the WHO-recommended first-line treatment for uncomplicated malaria, is 98% effective in producing adequate parasitological and clinical response (APCR) necessary for a child to be cured but is up to twenty times more costly than significantly less effective monotherapies, such as chloroquine and sulfadoxine-pyrimethamine [14, 15]. On account of high cost to individuals, artemisinin-based combinations are used to treat only 40% of under-five malaria cases in Nigeria [16]. Financial barriers especially hurt the poorest and most vulnerable Nigerians, who use ACT at about half the rate of the richest [16].

Prior studies of countries in sub-Saharan African have demonstrated that appropriate diagnosis and treatment are cost-effective interventions for case management of under-five malaria, but there are knowledge gaps regarding the cost-effectiveness of such interventions in Nigeria in particular, as well as how equitable such interventions would be [26]. Equity consideration in evidence-informed policymaking will be crucial to improving child health outcomes in Nigeria—with an exceptionally high under-five mortality rate amongst its poorest children (14% vs. 5% for the poorest and richest fifth, respectively), child health equity is among Nigeria’s most pressing public health challenges [27–29].

This study estimates the potential health and economic benefits of publicly financing case management of under-five malaria in Nigeria through the provision of government subsidies. Particularly, extended cost effectiveness analysis (ECEA) is applied to estimate intervention benefits across different socioeconomic groups. To account for constraints in government health budgets, the effects of multiple financing strategies that offer varying levels of coverage were investigated [30]. ECEA is an analytical technique that assesses health interventions across two main dimensions: health gains and financial risk protection afforded (Box 2) [31]. Moreover, ECEA disaggregates the effects of interventions across population strata of interest, allowing policymakers to identify which subgroups within a broader target population would benefit most from an intervention [31]. This study adds new value to existing cost-effectiveness research by for the first time investigating how the health and economic benefits of any malaria intervention would be distributed across socioeconomic lines in Nigeria, with the goal of informing policymakers in creating targeted, cost-effective interventions that help the most vulnerable people while preserving limited financial resources [32].

Box 1: The financial landscape of malaria treatment in Nigeria In observance of the United Nations Millennium Development Goals (MDGs) for 2000–2015, which aimed in part to eradicate poverty and reduce child mortality, Nigeria’s National Health Insurance Scheme piloted the Free Maternal and Child Health Program (FMCHP) in 2009 [17, 18]. Among other services, the FMCHP provided free malaria treatment to under-five children brought to public health facilities in 12 out of 36 states [18, 19]. Introduction of the FMCHP coincided with national reductions in child mortality, indicating potential efficacy of the programme [18]. However, the FMCHP ended in 2015 with the conclusion of the MDGs, as states were unable to sustain necessary funding [18]. As a result, most Nigerian’s currently pay for malaria treatment out of pocket (OOP) [10].

The OOP cost of treating under-five malaria accounts for nearly half of all household medical expense in Nigeria, significantly contributing to catastrophic health expenditure (CHE) in the poorest households [20]. Generally defined as medical expenditure exceeding 10% of annual income, CHE not only leaves individuals unable to pay for future essential health services but often leads to cycles of poverty [21, 22]. Globally, CHE pushes nearly 100 million people into poverty per year, exacerbating child health inequalities. Offering financial risk protection, or protection against illness-related impoverishment resulting from CHE, will be essential to make progress toward several of the WHO’s Sustainable Development Goals (SDGs) for 2016–2030 [23].

To that end, scaling up programmes like the FMCHP for the provision of effective malaria treatment could incentivize care-seeking behaviour and increase service use of the most effective therapies, which may have a positive, pro-poor impact on child health in Nigeria [19, 23, 24]. Such an intervention would contribute progress toward several SDGs, namely SDG 1 (reducing poverty), SDG 3 (ensuring good health and well-being at all ages), and SDG 10 (reducing inequality within societies) [1, 24]. Financing under-five malaria treatment through government subsidies would also offer protection against a major source of CHE in Nigeria, which will help achieve SDG target 3.8: achieving Universal Health Coverage (UHC) that ensures access to essential health-care services and access to safe, effective, quality and affordable essential medicines for all [24, 25].

Box 2: ECEA (extended cost-effectiveness analysis) ECEA is a novel analytical technique that quantifies the outcomes of interventions across four dimensions: health benefits, economic benefits, financial risk protection afforded (FRP), and cost of implementation. ECEA also disaggregates intervention effects across population strata. For example, ECEA can take into account the differences between income groups, regional and geographical distributions, rural and urban settings, ethnic groups, sex, marginalized populations, and other groups where health and financial outcomes may vary substantially [31]. As Nigeria has high levels of socioeconomic inequality, this study uses ECEA to determine the distributional effects of malaria interventions across socioeconomic lines in Nigeria by disaggregating the target population into five wealth quintiles (Q1–Q5, in ascending order of income). Health benefits, economic benefits, FRP afforded, and cost of implementation were estimated separately for each quintile.

ECEA estimates health benefits by quantifying either deaths averted, disability-adjusted life years (DALYs) averted, or quality-adjusted life years (QALYs) gained. ECEA estimates economic benefits by quantifying the total out-of-pocket (OOP) health expenditure averted by individuals benefiting from an intervention. Financial risk protection, a measure of the extent to which beneficiaries of an intervention are protected from illness-related impoverishment, can be measured in cases of catastrophic health expenditure (CHE) averted. A case of CHE is generally defined as an individual spending more than 10% of total annual income or 40% of annual non-food expenditure on health-related costs.

## Methods

An extended cost-effectiveness analysis (ECEA) was done using a decision tree model created with TreeAge Pro Healthcare software, Version 2020 R2. Using demographic and epidemiological data from published literature and unpublished costing data as parameters, the health and economic effects of three different intervention scenarios were quantified over a year of implementation, disaggregated across five wealth quintiles (Q1–Q5 in ascending order of wealth index). The intervention outcomes estimated were the number of under-five deaths averted, OOP expenditure averted, cases of catastrophic health expenditure (CHE) averted, and the cost of implementation.

### Interventions

Case management of under-five malaria incurs direct medical costs (consultation, appropriate diagnosis, medical supplies, drugs), non-medical costs (food on the way to the health facility, transportation, other non-medical supplies and services), and indirect costs (income forgone in productive time lost to caregiving) [33–35]. Non-medical and indirect costs often represent a high proportion of total expense associated with case management, especially for severe cases requiring inpatient hospitalization and significant time spent away from work for caregiving [19, 36].

While the FMCHP subsidized direct medical costs of treatment, off-setting non-medical and indirect costs will more effectively mitigate the economic burden of treating under-five malaria and could further incentivize service use of effective treatment [19]. To consider variable financing capacity, three intervention scenarios were modelled: (1) a 50% subsidy of direct medical costs (50% DMC), (2) a full subsidy of direct medical costs (full DMC), and (3) a full subsidy of direct medical costs in addition to compensating individuals for non-medical and indirect costs through a voucher system (full DMC + NMC + IC).

Greater increase in treatment coverage was assumed for the interventions covering a greater proportion of total costs, as they are likely to incentivize better care-seeking behaviour [37]. A relatively higher increase in treatment coverage in poorer quintiles was also assumed, based on findings indicating that FMCHP clinics were disproportionately serviced by poorer and more disease-burdened socioeconomic groups [18]. Respectively per quintile, modelled were: a 2.5, 2, 1.5, 1, and 0.5% point increase in treatment coverage for the 50% DMC subsidy; a 5, 4, 3, 2, and 1% point increase in treatment coverage for the full DMC subsidy; and a 10, 8, 6, 4, and 2% percentage point increase in treatment coverage for the full DMC + NMC + IC subsidy (Table 1).

**Table 1:** Summary of model parameters for Extended Cost-Effectiveness Analysis (ECEA)

	Parameter	Value for each wealth quintile (Q1–Q5) if applicable	References and notes
Demographics	Number of under-five children in Nigeria	Q1 = 8,822,526 Q2 = 7,737,789 Q3 = 6,436,105 Q4 = 5,806,958 Q5 = 5,185,042	Authors’ calculation using national population size, median household size, and number of under-fives per households from [16]
Epidemiology	Treatment sought for under-5 malaria (%)	Q1 = 67.8 Q2 = 70.4 Q3 = 72.4 Q4 = 79.1 Q5 = 85.2	[16]
	Annual cases of under-five malaria in Nigeria	Q1 = 6,960,185 Q2 = 6,468,963 Q3 = 5,261,275 Q4 = 3,361,056 Q5 = 1,318,521	Authors’ calculation using prevalence data from [16]
	Cumulative annual incidence of uncomplicated under-five malaria (%)	Q1 = 77.1 Q2 = 81.9 Q3 = 80.2 Q4 = 57.0 Q5 = 25.2	Authors’ calculation using treatment-seeking behaviour and probability of disease progression to severe from [16] and [53]
	Cumulative annual incidence of severe under-five malaria (%)	Q1 = 1.8 Q2 = 1.7 Q3 = 1.6 Q4 = 0.8 Q5 = 0.3	Authors’ calculation using treatment-seeking behaviour and probability of disease progression to severe from [16] and [53]
	Treatment coverage increase for 50% DMC subsidy (percentage point)	Q1 = 2.5 Q2 = 2 Q3 = 1.5 Q4 = 1 Q5 = 0.5	Authors’ assumption
	Treatment coverage increase for full DMC subsidy (percentage point)	Q1 = 5 Q2 = 4 Q3 = 3 Q4 = 2 Q5 = 1	Authors’ assumption
	Treatment coverage increase for full DMC + NMC + IC subsidy (percentage point)	Q1 = 10 Q2 = 8 Q3 = 6 Q4 = 4 Q5 = 2	Authors’ assumption
	ACT prescribed in those seeking treatment (%)	Q1 = 46.6 Q2 = 51.5 Q3 = 52.5 Q4 = 53.1 Q5 = 61	[16]
Treatment	ACT efficacy (%)	98.3	[14]
	Adherence to treatment for uncomplicated cases (%)	Q1 = 66 Q2 = 71 Q3 = 76 Q4 = 81 Q5 = 86	Authors’ assumption using overall estimate of adherence across all wealth indices from [40]
	Efficacy of ACT for uncomplicated cases given non-adherence as a proportion of theoretical efficacy (%)	94.7	[54]
	Non-ACT efficacy (%)	63	Authors’ calculation using efficacy of chloroquine and all other non-ACTs from [6]
	Probability that untreated case progresses to severe (%)	7	Calibrated with low estimates from [53]
	Probability that treatment failure progresses to severe (%)	2	[55]
	CFR of untreated severe malaria (%)	45	Calibrated with low estimates from [52]
	CFR of treated severe malaria (%)	4.9	[56]
	CFR of untreated uncomplicated malaria (%)	0.1	Authors’ assumption based on [57]
Costing (2020 $US)	Outpatient OOP direct medical costs per case, ACTs used	7.98	Authors’ calculation using forthcoming data from multi-facility Duke costing study (see Additional file 1 Section I, Table S8)
	Outpatient OOP direct medical costs per case, non-ACTs used	6.29	Authors’ calculation using [6]**.** and forthcoming data from multi-facility Duke costing study (see Additional file 1 Section I, Tables S8 and S9)
	Outpatient OOP direct non-medical costs per case	2.26	Authors’ calculation using forthcoming data from multi-facility Duke costing study (see Additional file 1 Section I, Table S10)
	Outpatient OOP indirect costs per case	Q1 = 0.49 Q2 = 0.78 Q3 = 1.14 Q4 = 1.70 Q5 = 3.16	Authors’ calculation using data on daily consumption and days spent caregiving from [58]
	Inpatient OOP direct medical costs per case	39.25	Authors’ calculation using forthcoming data from multi-facility Duke costing study (see Additional file 1 Section I, Table S8)
	Inpatient OOP direct non-medical costs per case	4.16	Authors’ calculation using forthcoming data from multi-facility Duke costing study (see Additional file 1 Section I, Table S10)
	Inpatient OOP indirect costs per case	Q1 = 2.98 Q2 = 4.75 Q3 = 6.91 Q4 = 10.36 Q5 = 19.26	Authors’ calculation using data on daily consumption and days spent caregiving from [58, 59]
	OOP indirect cost of non-treatment per untreated case (uncomplicated)	Q1 = 2.45 Q2 = 3.9 Q3 = 5.67 Q4 = 8.49 Q5 = 15.79	Authors’ calculation using data on daily consumption and days spent caregiving from [58, 59]
	OOP indirect cost of non-treatment per case (severe)	Q1 = 4.89 Q2 = 7.79 Q3 = 11.34 Q4 = 16.99 Q5 = 31.58	Authors’ calculation using data on daily consumption and days spent caregiving from [58, 59]
	Outpatient cost of implementation per case, ACTs used	10.50	Authors’ calculation using estimates of OOP expenditure as a percentage of total health expenditure in Nigeria [43]
	Outpatient cost of implementation per case, non-ACTs used	8.28	Authors’ calculation using estimates of OOP expenditure as a percentage of total health expenditure in Nigeria [43]
	Inpatient cost of implementation per case	51.65	Authors’ calculation using estimates of OOP expenditure as a percentage of total health expenditure in Nigeria [43]
	Nigeria GNI	2030	[60]
	Nigeria Gini Index	35.1	[61]

*DMC* direct medical cost, *NMC* non-medical cost, *IC* indirect cost, *ACT* artemisinin-based combination therapy, *CFR* case-fatality rate, *OOP* out-of-pocket, *GNI* gross national income

### Model parameters

Parameters were disaggregated by wealth quintile using empirical data from the 2018 Nigeria Demographic and Health Survey (DHS) when possible and estimated when disaggregated data were not available (Table 1) [16]. Calculations for estimating select model parameters by quintile are described in detail in Additional file 1 Section I. Estimated parameters were calibrated so the model accurately estimated the reported number of annual under-five malaria deaths in Nigeria in a base case scenario.

### Model flow

Using TreeAge Pro Healthcare decision analysis software, one tree for each wealth quintile (Q1–Q5, in ascending order of wealth) was created. Each decision tree is identical in structure, but parameter values differ based on wealth quintile. The entry point for each tree is a case of under-five malaria. Annual cases of under-five malaria per quintile were estimated using data from the 2019 World Malaria Report, the U.S. President’s Malaria Initiative Nigeria Malaria Operational Plan for 2020, and the 2018 Nigeria DHS [1, 16, 38].

**Fig. 1:**
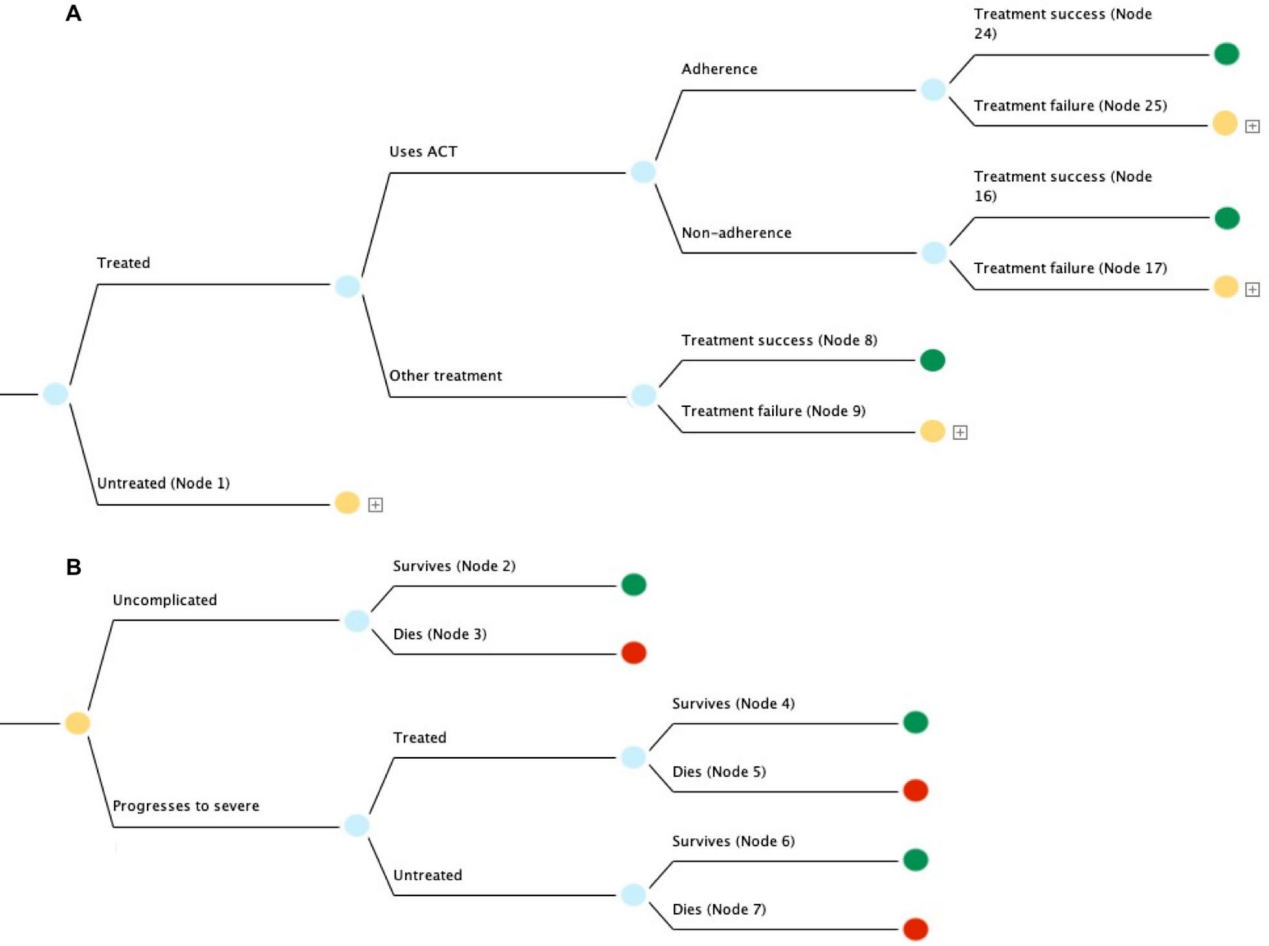
**A **Decision tree used to model annual deaths and OOP expenditure associated with treating under-five malaria in Nigeria. 23 million annual cases were simulated. Identical trees were used for each wealth quintile (Q1–Q5) but with different parameters for costs, mortality, and case load. Green terminal nodes represent survival and orange nodes feed into subtrees representing cases that are either untreated or where treatment failure occurs, where disease prognosis may progress to severe (Nodes 1, 9, 17, and 25). **B **Subtree modelling under-five malaria cases that are either untreated or where treatment failure occurs. Green and red terminal nodes respectively represent survival and death

The model’s first chance node splits into clinically treated and untreated cases (Fig. 1A). The probability that a case is clinically treated was proxied by the proportion of febrile under-fives for whom treatment is sought, available from the 2018 Nigeria DHS. This approximation was based on the fact that fever in under-fives is a relatively good indicator of malaria in endemic countries [39]. Treated cases split into those treated with ACT and without ACT (e.g., chloroquine, sulfadoxine-pyrimethamine), since ACT is the recommended standard first-line treatment but not always used. As the efficacy of ACT is highly dependent on adherence to treatment course, cases treated with ACT split into those both with and without proper adherence [40].

Both adherent and non-adherent cases were split into treatment success (defined as adequate parasitological and clinical response—i.e., clearance of parasitaemia) and treatment failure. Cases treated with non-artemisinin-based combinations were split directly into treatment success and failure on account of low overall treatment success of non-artemisinin-based combinations [41]. Treatment success results in a green terminal node representing survival (nodes 8, 16, and 24).

The orange nodes labeled Node 1, 9, 17, and 25 represent untreated cases or treatment failure; accordingly, these nodes branch into trees modelling progression to severe disease (Fig. 1B). The trees emanating from nodes 1, 9, 17 and 25 are identical in structure but have parametric differences according to the specific therapeutic scenario, discussed in more detail in Additional file 1 Section II. Node 1 is displayed in Fig. 1B, initially splitting into cases that remain uncomplicated or progress to severe. Uncomplicated cases split into survival and death, respectively resulting in terminal nodes 2 (green) and 3 (red). Severe cases split into cases that are treated and untreated. It was assumed that probability of seeking treatment for severe cases that were initially untreated was the same as the probability of initially seeking treatment for uncomplicated cases. Treated and untreated cases each split into survival and death, resulting in terminal nodes 4, 5, 6 and 7.

Using the TreeAge cost-effectiveness setting, cost and effectiveness values were assigned at all terminal nodes. Nodes representing survival and death were respectively assigned effectiveness values of 1 and 0. For each scenario, OOP costs were assigned in 2020 United States Dollars (USD). Uncomplicated and severe cases were respectively assumed to incur outpatient and inpatient costs, a standard assumption in malaria modelling studies [22]. Additionally, untreated cases were assigned the indirect cost of non-treatment (i.e., income foregone by the caregiver in order to take care of a sick child who does not receive treatment); this indirect cost was estimated separately for each quintile by multiplying the average length of untreated illness with daily wage per quintile.

For example, terminal node 24, which corresponds to uncomplicated cases treated with ACT, was assigned a total case cost that includes outpatient direct medical costs, non-medical costs, and indirect costs (Fig. 1A). Terminal node 2, corresponding to untreated uncomplicated cases, was assigned only the indirect cost of non-treatment for uncomplicated cases (Fig. 1B). Terminal node 4, corresponding to cases that were initially untreated but treated upon progressing to severe, was assigned a total case cost that included inpatient direct medical costs, non-medical costs, and indirect costs. Costing assignments for all terminal nodes are described fully in Additional file 1 Section II.

For the 50% DMC intervention, direct medical costs were set at 50% of base case values. For the full DMC intervention, direct medical costs were set at 0. For the full DMC + NMC + IC intervention, all costs were set at 0. About 23 million annual cases under-five cases were simulated across quintiles, a number corroborated by prior malaria modelling studies in Nigeria and data from the Institute of Health Metrics and Evaluation (IHME) Global Burden of Disease database [6, 42]. For each modelled scenario (base case plus three interventions), surviving cases and annual OOP expenditure were generated stochastically with Monte Carlo simulations using the TreeAge microsimulation tool. For intervention scenarios, increased coverage was simulated by increasing treatment probability at the first chance node (Fig. 1). OOP expenditure and surviving cases per intervention were compared against the outcomes of the base case scenario in which treatment coverage rates were set as the status quo and no subsidy was applied to treatment costs. Additionally, the incremental OOP expenditure averted between each intervention scenario was compared.

Individual cases of catastrophic health expenditure (CHE) were estimated using a simple disease model, described in Additional file 1 Section IV [31]. Cases of CHE were calculated using a threshold of 10% of annual per capita income, and CHE attributable to outpatient and inpatient care were estimated separately.

### Cost of implementation

For each intervention, the government cost of implementation was estimated using the same decision tree that was used to model deaths and OOP expenditure, but costing parameters were changed to reflect government costs [22]. In Nigeria, OOP spending represents 76% of total current health expenditure while government spending represents 24% [43]. The cost of implementing each intervention was modelled accordingly. For the full DMC intervention, where the total direct medical costs of treatment are fully subsidized, the government cost of implementation per case was estimated by dividing the OOP direct medical cost per case by 76%. For the 50% DMC intervention, the government cost of implementation per case was set as 50% of this value. For the full DMC + NMC + IC intervention, the government cost of implementation per case was set as the sum of total direct medical costs per case (i.e., OOP cost divided by 76%) plus OOP non-medical and indirect costs per case. See Table 1 for estimated government costs per case.

### Sensitivity analysis

Uncertainty in model outcomes (deaths, OOP expenditure, and CHE averted) was quantified using univariate sensitivity analysis. Each model parameter was changed to reflect a high value and low value scenario (respectively 20% higher and 20% lower than the base value). The relative impact of parameter variability was quantified by taking the percent difference between the model outcomes of high value scenarios and base value scenarios and doing the same for low value scenarios and base value scenarios. Uncertainty was estimated for broad intervention effects across all quintiles, averaged across all intervention scenarios unless otherwise noted in results.

### Optimization analysis

To determine how malaria funds could be optimally allocated across different socioeconomic quintiles using different combinations of interventions, ECEA results were used as inputs for an optimization framework. The inputs used were benefits per dollar spent on each intervention in each quintile. The objective function minimized total policy cost given constraints on distribution of intervention outcomes. This approach was taken such that both deaths and cases of CHE averted could be considered within a single framework. Under this scenario, three kinds of constraints were added: first, it was required that policymakers would want a 25% reduction in malaria mortality in Nigeria. According to the World Health Organization (WHO) World Malaria Report 2019, there were about 100,000 annual malaria deaths in Nigeria in 2019, so an overall constraint on the objective function was total deaths averted ≥ 25,000 [1]. Similarly, the second overall constraint was that total cases of CHE averted ≥ 25,000. The third constraint was that malaria mortality should be reduced by at least 25% within each quintile; this was necessary to ensure proportional spread of benefits across all quintiles while still allowing the most affected socioeconomic groups to benefit most when measured in absolute numbers. Baseline deaths per quintile were modeled using baseline inputs in the decision tree. See Additional file 1 Section IX for the complete optimization methodology.

## Results

### Base case mortality and economic burden of under-five malaria in Nigeria

In the base case scenario without intervention, the model estimated a total of 93,734 annual under-five malaria deaths, 8,637 annual individual cases of CHE, and US$211.9 million in annual OOP expenditure because of treating under-five malaria in Nigeria (Table 2). The poorest two quintiles accounted for 66% of mortality, 76% of CHE, and 53% of OOP expenditure. Apart from quintile 1, cases of CHE were exclusively attributable to the cost of inpatient hospitalization.

**Table 2:** Base case annual under-five malaria health and economic indicators in Nigeria

Wealth quintile	Under-five deaths (thousands)	OOP expenditure (millions, US$)	Cases of CHE (thousands)
Q1	34.3	52.9	4.0
Q2	27.9	55.1	2.6
Q3	20.6	49.6	1.7
Q4	8.9	36.5	0.4
Q5	2.0	17.8	0
Total	93.7	211.9	8.6

### Deaths averted through interventions

**Fig. 2:**
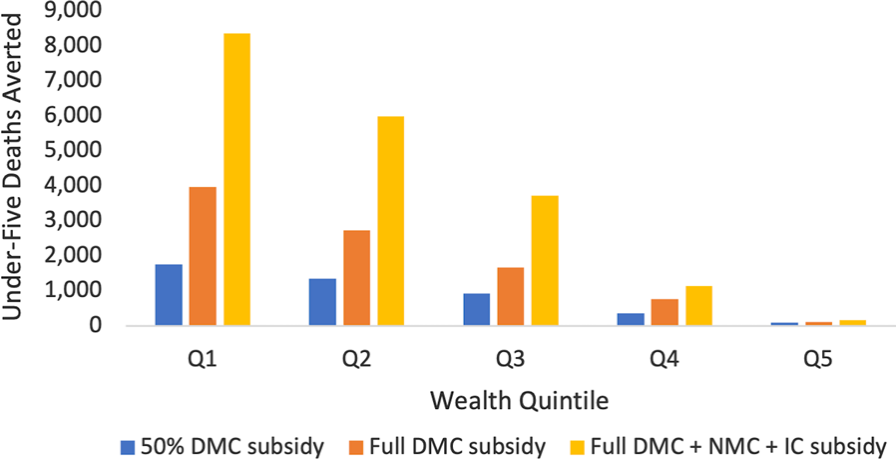
Annual under-five deaths averted through case management subsidies. Three different case management subsidies were modelled across socioeconomic lines. Deaths averted are concentrated among the poor

The 50% DMC, full DMC, and full DMC + NMC + IC subsidies respectively averted a total of about 4,500, 9,300, and 19,000 under-five deaths (Fig. 2). Across all intervention scenarios, health benefits were concentrated among the poor, with the poorest two quintiles accounting for 72% of all deaths averted and the richest quintile accounting for 1% of all deaths averted on average (see Additional file 1 Section VI, Fig. S3).

### OOP expenditure averted through interventions

**Fig. 3:**
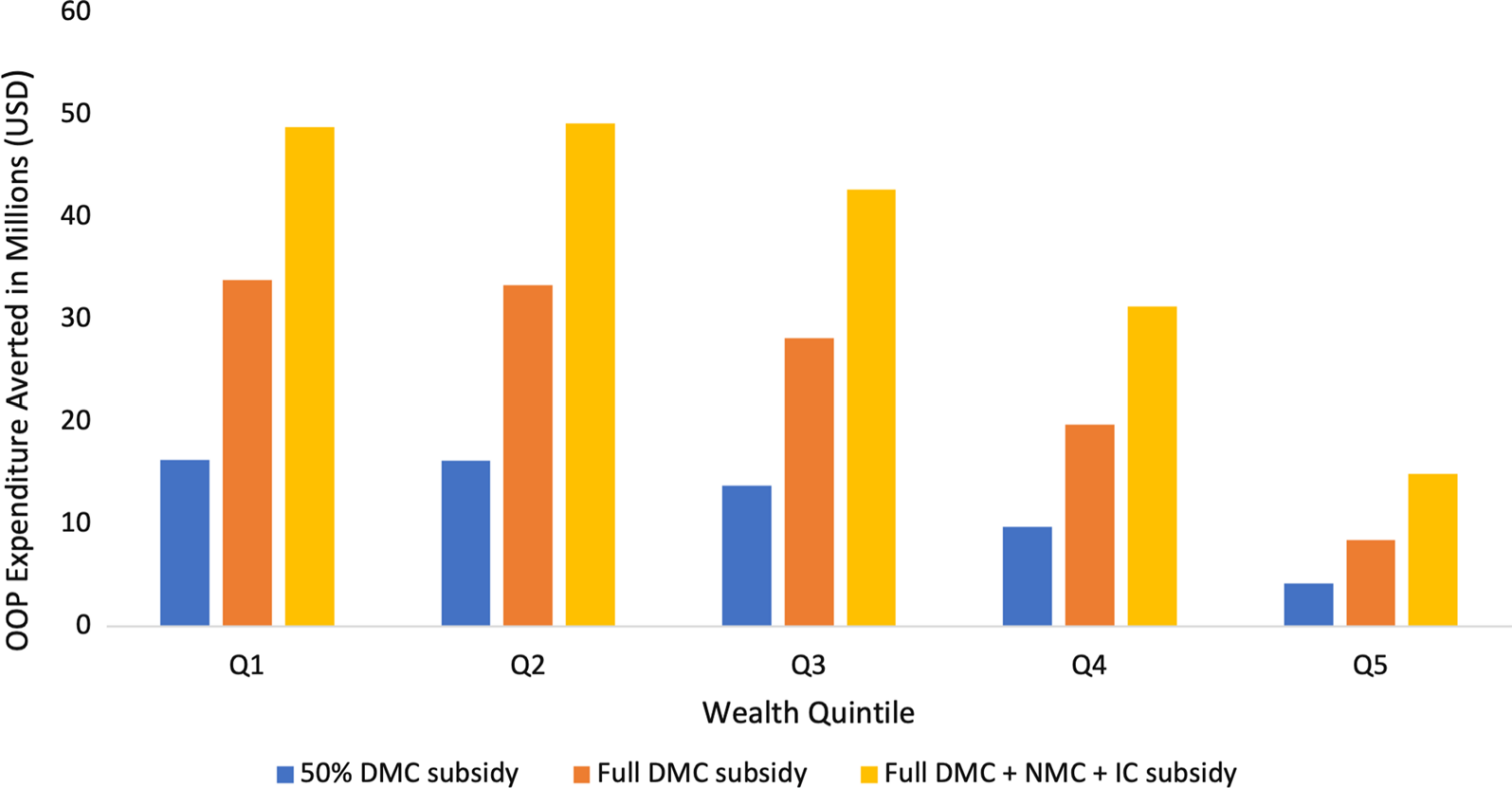
Annual OOP expenditure averted through case management subsidies. Three different case management subsidies were modelled across socioeconomic lines. OOP expenditure averted is concentrated among the poor

The 50% DMC, full DMC, and full DMC + NMC + IC subsidies respectively averted a total of US$60.3 million, US$123.7 million, and US$186.9 million in OOP expenditure across all quintiles (Fig. 3). In all intervention scenarios, benefits were concentrated among the poor, with the poorest two quintiles accounting for 55% and the richest quintile for accounting for 7% of total OOP expenditure averted on average (Additional file 1 Section VI, Fig. S4).

**Fig. 4:**
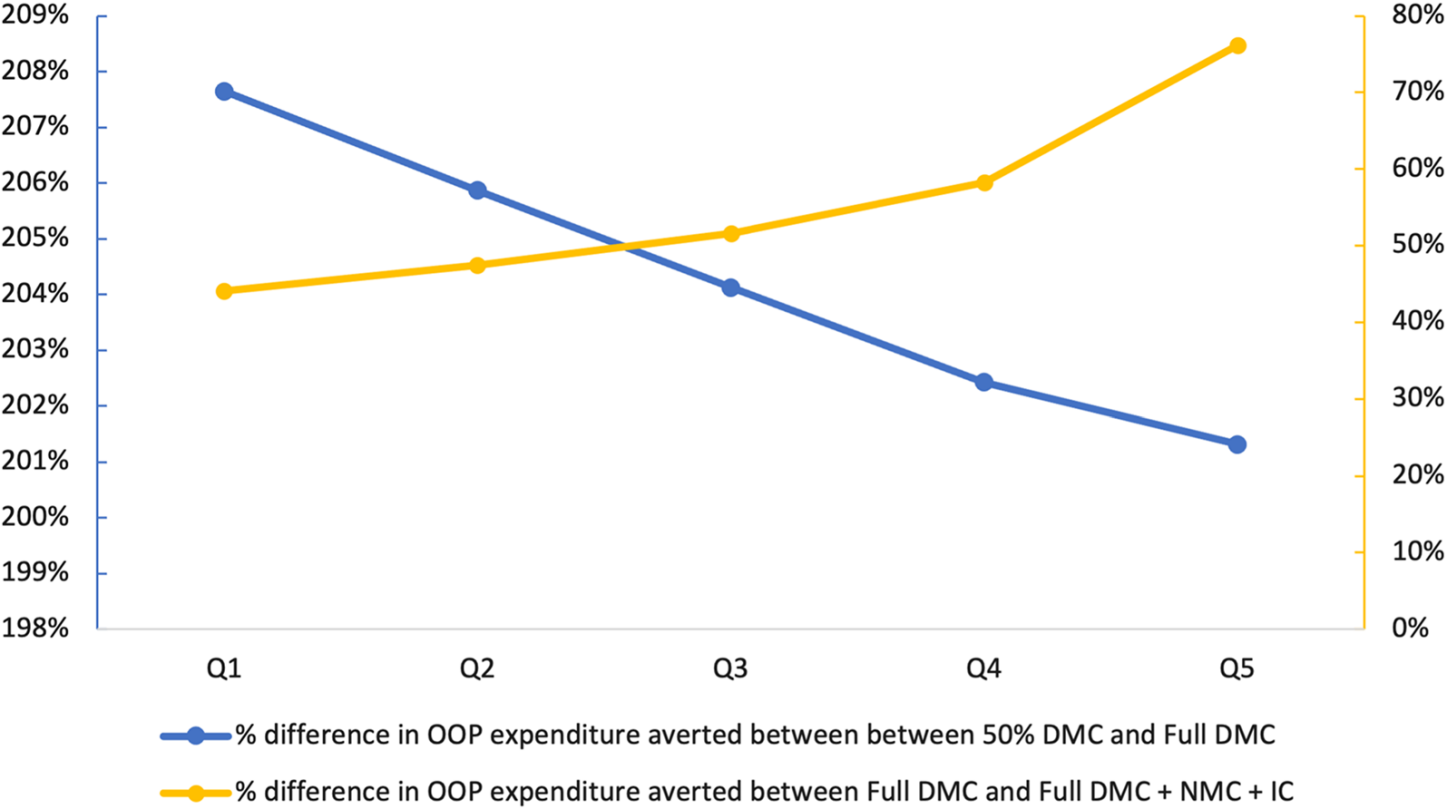
Incremental economic benefits of case management subsidies. For interventions subsidizing direct medical costs, incremental economic benefits are marginally greater for the poor than the wealthy (blue line). For interventions subsidizing nonmedical and indirect costs, incremental economic benefits are greater for the wealthy than the poor (yellow line)

In terms of OOP expenditure averted, the incremental benefits across interventions were greater between the 50% DMC and the full DMC subsidies than between the full DMC and full DMC + NMC + IC subsidies. On average across quintiles, the full DMC subsidy resulted in 204% more OOP expenditure averted than the 50% DMC subsidy, while the full DMC + NMC + IC subsidy resulted in 56% more OOP expenditure averted than the full DMC subsidy (Fig. 4). The incremental economic benefits of the full DMC subsidy were marginally greater for the poor than the wealthy, while the incremental economic benefits of the full DMC + NMC + IC subsidy were much greater for the wealthy than the poor.

### Financial risk protection afforded through interventions

**Fig. 5:**
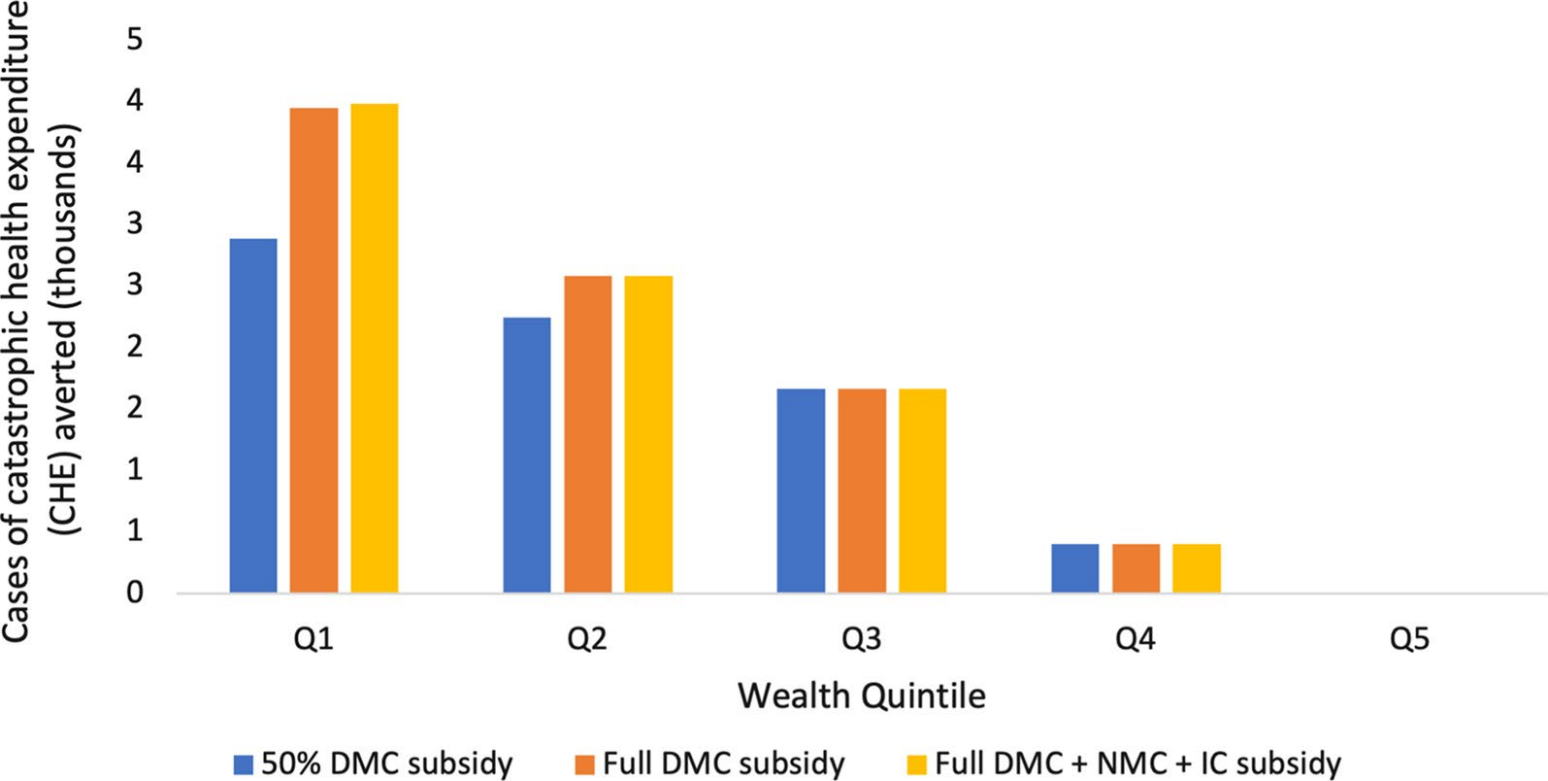
Annual financial risk protection afforded through case management subsidies. Annual under-five malaria related CHE averted by wealth quintile (Q1–Q5) in Nigeria by implementing three different interventions. CHE averted is concentrated among the poor

The 50% DMC, full DMC, and full DMC + NMC + IC subsidies respectively averted a total of 7,202, 8,604, and 8,637 annual cases of individual CHE (Fig. 5). Across all intervention scenarios, the poorest two quintiles accounted for 74% of all cases of CHE averted on average (Additional file 1 Section VI, Fig. S5), while quintile 5 experienced no CHE benefits. Only quintiles 1 and 2 experienced incremental benefits between the 50% DMC and the full DMC subsidies, with quintile 1 experiencing greater incremental benefits. Additionally, only quintile 1 experienced incremental benefits between the full DMC subsidy and the full DMC + NMC + IC subsidy, which were marginal.

### Cost of implementation

The 50% DMC, full DMC, and full DMC + NMC + IC subsidies would respectively cost the government US$90.5 million, US$179.1 million, and US$254.4 million to implement over one year (Table 3). Across all intervention scenarios, most expenditure would go toward treatment coverage for the poorest Nigerians, with the poorest two quintiles accounting for 56% of total intervention costs on average (Additional file 1 Section VI, Fig. S6).

**Table 3:** Cost of implementation (in millions, US$)

Wealth quintile	Q1	Q2	Q3	Q4	Q5	Total
50% DMC subsidy	25.7	24.7	20.4	13.9	5.8	90.5
Full DMC subsidy	51.1	48.9	40.3	27.4	11.5	179.1
Full DMC + NMC + IC subsidy	70.5	68.2	57.3	40.1	18.3	254.4

### Targeted subsidies: benefits per US$1 million invested in each quintile

Per US$1 million invested in each quintile, deaths and cases of CHE averted were greatest among the poor. For all interventions, investing US$1 million into either of the bottom two quintiles would avert more deaths and cases of CHE than investing broadly across all quintiles (Table 4). Per US$1 million invested in each quintile, OOP expenditure averted would be greater for wealthier quintiles; however, there would be a greater degree of equity in the intervention scenarios that do not subsidize nonmedical and indirect costs. For subsidies of nonmedical and indirect costs, wealthy quintiles would save significantly more OOP expenditure than poor quintiles per dollar spent by the government.

**Table 4:** Benefits per US$ 1 million invested in each quintile through targeted subsidies

Scenario	50% DMC subsidy			Full DMC subsidy			Full DMC + NMC + IC subsidy		
Wealth quintile	Deaths averted	Cases of CHE averted	OOP expenditure averted ($US)	Deaths averted	Cases of CHE averted	OOP expenditure averted ($US)	Deaths averted	Cases of CHE averted	OOP expenditure averted ($US)
Q1	68	112	634,966	78	77	664,423	118	56	692,694
Q2	55	91	656,198	56	53	682,355	88	38	721,303
Q3	46	81	676,488	42	41	699,007	65	29	745,170
Q4	26	29	702,522	28	15	721,404	28	10	780,206
Q5	17	0	726,646	10	0	737,800	10	0	816,586
All quintiles (broad subsidy)	50	80	666,372	52	48	690,530	76	34	734,889

### Sensitivity analysis

When changed by 20%, the most impactful model parameters on all ECEA outcomes on average were (1) the total number of annual under-five malaria cases and (2) initial treatment coverage. The number of under-five deaths averted was particularly impacted by (1) the probability that an untreated case progresses to severe and (2) the case fatality rate of untreated severe cases. OOP expenditure averted was more impacted by uncertainty in outpatient costs than inpatient costs, and uncertainty in direct medical costs was more impactful than uncertainty in nonmedical and indirect costs. The most impactful parameter on cases of CHE averted was inpatient direct medical cost. The cost of implementation was substantially more impacted by uncertainty in the costs of outpatient care than inpatient care. Combinations of parameters were also varied to determine their combined effect on ECEA outcomes. It was found that varying cases of malaria and initial treatment coverage together had a similar effect as varying initial treatment coverage alone in terms of deaths averted, but nearly doubled the effect of either parameter alone in terms of OOP expenditure averted. The effect on CHE averted and cost of implementation was similar to varying each parameter alone. Tornado charts and a summary of all sensitivity analysis results for all parameters is reported in the Additional file 1 Section VII.

### Optimization analysis

Given the constraints of at least 25,000 total deaths and cases of CHE averted, as well as a 25% reduction in deaths per quintile, the minimization function yielded a total policy cost of US$ 420 million (Table 5). Q1–Q5 accounted for 27%, 20%, 26%, 20%, and 7% of total policy cost, respectively. The full DMC subsidy was dominated across all quintiles. A combination of the 50% DMC and the Full DMC + NMC + IC subsidies was optimal for Q1, while the Full DMC + NMC + IC subsidy alone was optimal for Q2. For Q3-Q5, the 50% DMC subsidy dominated all other options.

**Table 5:** Optimal money allocation across quintiles and interventions

	Q1	Q2	Q3	Q4	Q5
50% DMC Subsidy	**$70 M**	Dominated	**$110 M**	**$85 M**	**$30 M**
Full DMC Subsidy	Dominated	Dominated	Dominated	Dominated	Dominated
Full DMC + NMC + IC Subsidy	**$45 M**	**$80 M**	Dominated	Dominated	Dominated

## Discussion

Extended cost-effectiveness analysis (ECEA) was used to model the health and economic effects of subsidizing case management of under-five malaria in Nigeria. The model’s estimated baseline mortality is comparable to figures reported by the WHO Severe Malaria Observatory and the IHME Global Burden of Disease database, which both report about 95,000 annual under-five malaria deaths in Nigeria [42, 44]. The model also estimates that Nigerians spend over US$200 million annually on case management of under-five malaria, which is a sizeable proportion of the reported US$700 million in annual OOP spending on malaria treatment, prevention, and other costs across all ages and wealth groups [1]. The model estimates that this high OOP expenditure results in over 8,600 annual individual cases of catastrophic health expenditure (CHE), 76% of which are concentrated among the poorest 40% of Nigerians (Table 2). These results emphasize the extent of child health inequity in Nigeria, stressing the need to employ an equity-focused approach to health policymaking that targets the poorest and most underserved populations rather than the mainstream, one-size-fits-all approach that may inadvertently favour the wealthy [26, 29]. This is especially important considering recent plateaus in donor funding of malaria control programmes, which may limit the scale of government interventions [35, 45].

A recent ECEA of malaria interventions in Ethiopia found that scaling up coverage of ACT would afford the greatest health benefits and financial risk protection relative to scaling up use of indoor residual spray, insecticide-treated bed nets, and a hypothetical vaccine [22]. Consistent with the present analysis, this study also found that the health and economic benefits of a treatment subsidy would be concentrated among the poor. Another ECEA found that compared to other interventions in Ethiopia, such as those addressing childhood diarrhoea and pneumonia, malaria interventions would see modest benefits, but this is likely due to the relatively low malaria burden in Ethiopia [46, 47]. The present analysis builds on the existing body of ECEA evidence for malaria interventions in sub-Saharan Africa by investigating different financing strategies that account for nonmedical and indirect costs and expanding geographic scope to Nigeria, the most malaria-burdened country in the world [1].

### Effects of broad subsidies

Larger case management subsidies would generally result in greater health and economic benefits. (Figs. 2, 3, 5). Across intervention scenarios, benefits would be concentrated among the poor, with the poorest 40% of children accounting for 72% of deaths averted, 55% of OOP expenditure averted, and 74% of all cases of CHE averted on average (Additional file 1 Section VI, Figs. S3–S5).

While a full DMC + NMC + IC subsidy would avert US$63.3 million more OOP expenditure than a full DMC subsidy, almost no difference was found in incremental cases of CHE averted between the two scenarios. In other words, financial risk protection benefits are limited to subsidizing direct medical costs of treatment. While non-medical and indirect costs may not themselves cause catastrophic health expenditure, compensating caregivers for these costs may still incentivize care-seeking because individuals in Nigeria often experience multiple different health problems in a given year (acute respiratory infection, pneumonia, HIV/AIDS, etc.), and the cumulative nonmedical and indirect costs could be catastrophic [48].

### Effects of targeted subsidies

Subsidies targeted to the poor would avert more deaths per dollar spent than non-targeted subsidies applied broadly across wealth groups. For example, in the full DMC + NMC + IC scenario, US$1 million invested into the poorest fifth of Nigerians would avert 118 deaths, while US$1 million invested broadly would avert 76 deaths, and US$1 million invested into the richest fifth would avert 10 deaths (Table 4). Furthermore, subsidies targeted to the poor would afford more financial risk protection per dollar spent than broad subsidies; in the full DMC + NMC + IC scenario, for example, investing US$1 million into the poorest fifth of Nigerians would avert 56 cases of CHE, compared to 34 cases averted if invested broadly and 0 cases averted if invested into the richest fifth (Table 4).

While targeted subsidies of direct medical costs would result in relatively equitable OOP savings across wealth groups, targeted subsidies of nonmedical and indirect costs would result in relatively more OOP savings for the wealthy (Fig. 4; Table 4). This is likely because higher income individuals incur higher indirect costs on account of productive time lost to caring for sick children. This assumption is supported by data from a study of direct and indirect costs of health services use in Nigeria that showed that compared to the general population, the poor had considerably lower indirect costs in terms of the value of time spent seeking care [49]. While wealthy quintiles incur greater indirect costs, these costs are less likely to be catastrophic [36].

### Optimization analysis

For the optimization scenario, across all wealth quintiles, fully subsidizing all direct medical costs was dominated by a 50% DMC subsidy or fully subsidizing medical, nonmedical, and indirect costs (Table 5). For the poorest quintile (Q1), the optimal strategy was to implement a two-pronged intervention with 60% of the Q1 funds allocated to a 50% DMC subsidy and 40% of the Q1 funds allocated to a Full DMC + NMC + IC subsidy. It will also require investments in Q2 to be 100% Full DMC + NMC + IC Subsidy, and investments in Q3, Q4, and Q5 to be allocated to the 50% DMC Subsidy only. Given that it might be challenging to specifically target households by wealth quintile, a policy maker could choose to target geographic regions or neighborhoods instead. For example, Nigeria’s 774 local government areas (LGAs) and six area councils can be ranked by average wealth, and the subsidies made available to the respective quintiles. As such, neighbourhoods that fall in the top richest quintiles will only receive the 50% DMC subsidy (about 20 million vouchers to Q3, 15 million vouchers to Q4, and 5 million vouchers to Q5). By contrast, neighbourhoods in the Q2 quintile will receive a total of about 10 million vouchers for the Full DMC + NMC + IC Subsidy, while the poorest neighbourhoods will receive a mix of 12 million vouchers for the 50% DMC and 3 million vouchers for the Full DMC + NMC + IC Subsidy (note: number of vouchers in this example was estimated by dividing total investment per quintile/unit cost per voucher for a given intervention). This smarter allocation of malaria intervention funds will improve efficiency and effectiveness of malaria control in Nigeria and is an example of using evidence to solve multicriteria decision making problems that policymakers face daily.

### Limitations

First, since the decision tree model is static, it was assumed that the case load of malaria would remain the same across the duration of the intervention course [50]. This assumption does not mirror the realities of transmissible diseases like malaria, but the dynamics of malarial transmission are unlikely to change significantly within a year of implementation, which is the projected time frame of the modelled scenarios. Furthermore, effective treatment has been shown to interrupt malaria transmission, so this policy could potentially move Nigeria closer to malaria elimination and lower costs in future years [51, 52]. Second, the model assumes that case management of uncomplicated malaria incurs outpatient health facility costs, but uncomplicated malaria is often treated in community settings in Nigeria [16]. The likely impact of this assumption is an overestimation of total OOP expenditure averted and total cost of implementation. Estimates of total financial risk protection, however, are relatively unaffected by this assumption because sensitivity analysis showed that CHE is most impacted by the cost of severe malaria cases requiring inpatient hospitalization (see Additional file 1 Section VII, Table S21). Third and most significantly, the methodology approximates initial treatment coverage rates as care-seeking behaviour for febrile under-fives, a common assumption made in modelling studies of malaria-endemic countries [22]. However, the aetiology of under-five fever can also include infection with HIV/AIDS, acute respiratory infection, anaemia, and pneumonia, so care-seeking for febrile under-fives may not reflect the true coverage rate for malaria treatment [48]. Initial treatment coverage is one of the most influential variables for estimation of all ECEA outcomes (average effect size of 24% when changed by 20%), so this is a key limitation (Additional file 1 Section VII, Table S21).

## Conclusions

Under-five malaria in Nigeria remains one of the biggest challenges to global child health. Stark health inequities between the rich and the poor necessitate the introduction of targeted interventions that benefit the most vulnerable. Targeted subsidization of case management of under-five malaria is a pro-poor intervention that leads to significant reductions in national under-five mortality and illness-related impoverishment, contributing progress to several of the Sustainable Development Goals including reducing poverty (SDG 1), ensuring good health and well-being at all ages (SDG 3), and reducing inequality within societies (SDG 10). This study provides recommendations for the adoption of smarter policies to improve efficiency and effectiveness of malaria control efforts in Nigeria.

## Supplementary Information

**Additional file 1**: Supporting information for methods and results of ECEA of case management of under-five malaria in Nigeria. The additional file contains detailed explanations of the methodology used to estimate certain non-empirical model parameters across wealth quintiles (e.g., caseload of under-five malaria). It also contains all ECEA and sensitivity analysis results, as well as full optimization analysis methodology.

## Data Availability

The demographic and epidemiological data that support the findings of this study are available from the Nigeria Demographic and Health Survey, https://dhsprogram.com/publications/publication-fr359-dhs-final-reports.cfm.

## Abbreviations

ACT: Artemisinin-based combination therapy
APCR: Adequate parasitological and clinical response
CFR: Case fatality rate
CHE: Catastrophic health expenditure
DHS: Demographic and health survey
DMC: Direct medical costs
ECEA: Extended cost-effectiveness analysis
FMCHP: Free Maternal and Child Health Program
IC: Indirect costs
IHME: Institute for Health Metrics and Evaluation
MDGs: Millennium Development Goals
NMC: Nonmedical costs
OOP: Out-of-pocket
SDGs: Sustainable Development Goals
UHC: Universal health coverage
USD: United States Dollar

## References

[1] WHO (2019). World malaria report 2019.

[2] WHO (2016). Global Technical Strategy for Malaria 2016–2030.

[3] WHO (2017). World malaria report 2017.

[4] WHO. Fact Sheet about Malaria. Geneva, World Health Organization. 2020. www.who.int/news-room/fact-sheets/detail/malaria. Accessed Dec 2020.

[5] Onwujekwe O, Uguru N, Etiaba E, Chikezie I, Uzochukwu B, Adjagba A (2013). The economic burden of malaria on households and the health system in Enugu State southeast Nigeria. PLoS ONE.

[6] Beargie SM, Higgins CR, Evans DR, Laing SK, Erim D, Ozawa S (2019). The economic impact of substandard and falsified antimalarial medications in Nigeria. PLoS ONE.

[7] Edelu BO, Ndu IK, Igbokwe O, Iloh ON (2018). Severe falciparum malaria in children in Enugu, South East Nigeria. Niger J Clin Pract.

[8] United States Embassy in Nigeria. Nigeria Malaria Fact Sheet. 2011. https://photos.state.gov/libraries/nigeria/231771/Public/December-MalariaFactSheet2.pdf. Accessed Dec 2020.

[9] United States Embassy in Nigeria, Nigeria Malaria Fact Sheet. 2013. http://photos.state.gov/libraries/nigeria/231771/Public/December-MalariaFactSheet2.pdf. Accessed Dec 2020.

[10] Adewemimo A, Kalter HD, Perin J, Koffi AK, Quinley J, Black RE (2017). Direct estimates of cause-specific mortality fractions and rates of under-five deaths in the northern and southern regions of Nigeria by verbal autopsy interview. PLoS ONE.

[11] Uneke C, Sombie I, Uro-Chukwu H, Johnson E (2019). Developing equity-focused interventions for maternal and child health in Nigeria: an evidence synthesis for policy, based on equitable impact sensitive tool (EQUIST). Pan Afr Med J.

[12] Ezenduka C, Falleiros D, Godman B (2013). Evaluating the treatment costs for uncomplicated malaria at a public healthcare facility in Nigeria and the implications. Pharmacoecon Open.

[13] Bhutta Z, Black R (2013). Global maternal, newborn, and child health-so near and yet so far. N Engl J Med.

[14] Thwing J, Eisele T, Steketee R (2011). Protective efficacy of malaria case management for preventing malaria mortality in children: a systematic review for the Lives Saved Tool. BMC Public Health.

[15] O'Connell KA, Gatakaa H, Poyer S, Njogu J, Evance I, Munroe E (2010). Got ACTs? Availability, price, market share and provider knowledge of anti-malarial medicines in public and private sector outlets in six malaria-endemic countries. Malar J.

[16] National Population Commission of Nigeria. Nigeria Demographic and Health Survey 2018. 2018. https://dhsprogram.com/publications/publication-fr359-dhs-final-reports.cfm. Accessed Dec 2020.

[17] Kumar S, Kumar N, Vivekadhish S (2016). Millennium development goals (MDGs) to sustainable development goals (SDGs): addressing unfinished agenda and strengthening sustainable development and partnership. Indian J Community Med.

[18] Onwujekwe O, Obi F, Ichoku H, Ezumah N, Okeke C, Ezenwaka U (2019). Assessment of a free maternal and child health program and the prospects for program re-activation and scale-up using a new health fund in Nigeria. Niger J Clin Pract.

[19] Ogbuabor D, Onwujekwe O (2018). Implementation of free maternal and child healthcare policies: assessment of influence of context and institutional capacity of health facilities in South-east Nigeria. Glob Health Action.

[20] Onwujekwe O, Hanson K, Uzochukwu B, Ichoku H, Ike E, Onwughalu B (2010). Are malaria treatment expenditures catastrophic to different socio-economic and geographic groups and how do they cope with payment? A study in southeast Nigeria. Trop Med Int Health.

[21] Acharya S, Lin V, Dhingra N (2018). The role of health in achieving the sustainable development goals. Bull World Health Organ.

[22] Assebe LF, Kwete XJ, Wang D, Liu L, Norheim OF, Jbaily A (2020). Health gains and financial risk protection afforded by public financing of selected malaria interventions in Ethiopia: an extended cost-effectiveness analysis. Malar J.

[23] Verguet S, Woldemariam AT, Durrett WN, Norheim OF, Kruk ME (2017). Is the sustainable development goal target for financial risk protection in health realistic?. BMJ Glob Health.

[24] WHO. Sustainable Development Goals. Geneva: World Health Organization; 2019. https://www.who.int/health-topics/sustainable-development-goals. Accessed Dec 2020.

[25] Abiiro GA, De Allegri M (2015). Universal health coverage from multiple perspectives: a synthesis of conceptual literature and global debates. BMC Int Health Hum Rights.

[26] White MT, Conteh L, Cibulskis R, Ghani AC (2010). Costs and cost-effectiveness of malaria control interventions: a systematic review. Malar J.

[27] UNICEF. Narrowing the gaps: the power of investing in the poorest children. 2017. https://www.equist.info/files/general_files/GLB_3418glb-9355narrowing-the-gaps-2017.pdf. Accessed Nov 2020.

[28] Waters D, Theodoratou E, Campbell H, Rudan I, Chopra M (2012). Optimizing community case management strategies to achieve equitable reduction of childhood pneumonia mortality: an application of Equitable Impact Sensitive Tool (EQUIST) in five low- and middle-income countries. J Glob Health.

[29] Chao F, You D, Pedersen J, Hug L, Alkema L (2018). National and regional under-5 morality rate by economic status for low-income and middle-income countries: a systematic assessment. Lancet.

[30] Carrera C, Azrack A, Begkoyian G, Pfaffmann J, Ribaira E, O'Connell T (2012). The comparative cost-effectiveness of an equity-focused approach to child survival, health, and nutrition: a modelling approach. Lancet.

[31] Verguet S, Kim J, Jamison D (2016). Extended cost-effectiveness analysis for health policy assessment: a tutorial. Pharmacoeconomics.

[32] Scott N, Hussain SA, Martin-Hughes R, Fowkes FJI, Kerr CC, Pearson R (2017). Maximizing the impact of malaria funding through allocative efficiency: using the right interventions in the right locations. Malar J.

[33] Houben CH, Fleischmann H, Gückel M (2013). Malaria prevalence in north-eastern Nigeria: a cross-sectional study. Asian Pac J Trop Med.

[34] Hailu A, Lindtjørn B, Deressa W, Gari T, Loha E, Robberstad B (2017). Economic burden of malaria and predictors of cost variability to rural households in south-central Ethiopia. PLoS ONE.

[35] Arrow KJ, Panosian C, Gelband H. The human and economic burden of malaria. In: Institute of Medicine (US) Committee on the Economics of Antimalarial Drugs (eds). Saving lives, buying time: economics of malaria drugs in an age of resistance. Washington (DC): National Academies Press (US); 2004. https://www.ncbi.nlm.nih.gov/books/NBK215634/. 25009879

[36] El-Houderi A, Constantin, J Castelnuovo, Sauboin, C. Economic and resource use associated with management of malaria in children aged <5 years in Sub-Saharan Africa: a systematic literature review. MDM Policy Pract. 2019;4:2381468319893986. 10.1177/2381468319893986 PMC6927205 31903421

[37] Cohen JL, Yadav P, Moucheraud C, Alphs S, Larson PS, Arkedis J (2019). Do price subsidies on artemisinin combination therapy for malaria increase household use? Evidence from a repeated cross-sectional study in remote regions of Tanzania. PLoS ONE.

[38] U.S. president’s malaria initiative Nigeria malaria operational plan FY 2020. www.pmi.gov (2020). Accessed Dec 2020.

[39] Stresman G, Sepulveda N, Fornace K, Grignard L, Mwesigwa J, Achan J (2020). Association between the proportion of *Plasmodium falciparum* and *Plasmodium vivax* infections detected by passive surveillance and the magnitude of the asymptomatic reservoir in the community: a pooled analysis of paired health facility and community data. Lancet Infect Dis.

[40] Yakasai AM, Hamza M, Dalhat MM, Bello M, Gadanya MA, Yaqub ZM (2015). Adherence to artemisinin-based combination therapy for the treatment of uncomplicated malaria: a systematic review and meta-analysis. J Trop Med.

[41] Antony HA, Parija SC (2016). Antimalarial drug resistance: an overview. Trop Parasitol.

[42] IHME GBD Results Tool. 2019. http://ghdx.healthdata.org/gbd-results-tool. Accessed Dec 2020.

[43] Aregbeshola BS, Khan SM (2018). Out-of-pocket health-care spending and its determinants among households in Nigeria: a national study. J Public Health.

[44] Nigeria severe malaria facts. 2020. https://www.severemalaria.org/countries/nigeria. Accessed Nov 2020.

[45] Korenromp E, Mahiane, Hamilton M, Pretorius C, Cibulskis R, Lauer J, et al. Malaria intervention scale-up in Africa: effectiveness predictions for health programme planning tools, based on dynamic transmission modelling. Malar J. 2016;15:417. 10.1186/s12936-016-1461-9 PMC4991118 27538889

[46] Pecenka CJ, Johansson KA, Memirie ST, Jamison DT, Verguet S (2015). Health gains and financial risk protection: an extended cost-effectiveness analysis of treatment and prevention of diarrhoea in Ethiopia. BMJ Open.

[47] Verguet S, Olson ZD, Babigumira JB, Desalegn D, Johansson KA, Kruk ME (2015). Health gains and financial risk protection afforded by public financing of selected interventions in Ethiopia: an extended cost-effectiveness analysis. Lancet Glob Health.

[48] Enya VNV, Idika N, Mafe AG, Akinside KN, Smith SI, Agomo PU (2014). Aetiology of fever among under fives in Lagos, Nigeria. BMC Infect Dis.

[49] Dauda DS, Ugaz S, Silfverberg D, Ogundipe A, Dutta A (2019). Assessment of direct fees and indirect costs for people seeking HIV services in Nigeria.

[50] Lugnér AK, Mylius SD, Wallinga J (2010). Dynamic versus static models in cost-effectiveness analyses of anti-viral drug therapy to mitigate an influenza pandemic. Health Econ.

[51] White NJ (2008). The role of anti-malarial drugs in eliminating malaria. Malar J.

[52] WHO (2015). Guidelines for the treatment of malaria.

[53] Lubell Y, Staedke SG, Greenwood BM, Kamya MR, Molyneux M, Newton PN (2011). Likely health outcomes for untreated acute febrile illness in the tropics in decision and economic models; a Delphi survey. PLoS ONE.

[54] Challenger JD, Bruxvoort K, Ghani AC, Okell LC (2017). Assessing the impact of imperfect adherence to artemether-lumefantrine on malaria treatment outcomes using within-host modelling. Nat Commun.

[55] Lubell Y, Dondorp A, Guerin PJ, Drake T, Meek S, Ashley E, Day NP, White NJ, White LJ (2014). Artemisinin resistance - modelling the potential human and economic costs. Malar J.

[56] Graham H, Bakare AA, Ayede AI, Oyewole OB, Gray A, Neal E, Qazi SA, Duke T, Falade AG (2020). Diagnosis of pneumonia and malaria in Nigerian hospitals: a prospective cohort study. Pediatr Pulmonol.

[57] Olliaro P (2008). Mortality associated with severe *Plasmodium falciparum* malaria increases with age. Clin Infect Dis.

[58] General Household Survey, Panel 2015–2016, Wave 3. Nigeria National Bureau of Statistics (NBS). 2016. https://microdata.worldbank.org/index.php/catalog/2734. Accessed Dec 2020.

[59] Hennessee I, Chinkhumba J, Briggs-Hagen M, Bauleni A, Shah MP, Chalira A, Moyo D, Dodoli W, Luhanga M, Sande J, Ali D, Gutman J, Lindblade KA, Njau J, Mathanga DP (2017). Household costs among patients hospitalized with malaria: evidence from a national survey in Malawi, 2012. Malar J.

[60] The World Bank: Gini index (World Bank estimate). https://data.worldbank.org/indicator/SI.POV.GINI (2020). Accessed Dec 2020.

[61] The World Bank: Nigeria. https://data.worldbank.org/country/NG (2020). Accessed Dec 2020.

